# Tris[6-meth­oxy-2-(phenyl­iminiometh­yl)phenolato]-κ^4^ *O*,*O*′;κ*O* ^1^-tris­(thio­cyanato-κ*N*)samarium(III)

**DOI:** 10.1107/S1600536811016205

**Published:** 2011-05-07

**Authors:** Guo-Di Ge, Jin-Bei Shen, Guo-Liang Zhao

**Affiliations:** a College of Chemistry and Life Science, Zhejiang Normal University, Jinhua 321004, Zhejiang, People’s Republic of China; b College of Chemistry and Life Sciences, Zhejiang Normal University, Jinhua, Zhejiang 321004, People’s Republic of China, and, Zhejiang Normal University Xingzhi College, Jinhua, Zhejiang 321004, People’s Republic of China

## Abstract

In the crystal structure of title compound, [Sm(NCS)_3_(C_14_H_13_NO_2_)_3_], two of the zwitterionic Schiff base 6-meth­oxy-2-(phenyl­iminiometh­yl)phenolate ligands coordinate to the Sm^III^ atom in a bidentate fashion *via* the phenolate and meth­oxy O atoms. The third Schiff base ligand is monodentate, binding only through the phenolate O atom. The coordination sphere of the eight-coordinate Sm atom is completed by the three independent thio­cyanate ions binding through their N atoms, affording a square-anti­prismatic geometry. An S atom of one of the thio­cyanate anions is disordered over two sites in a 0.85:0.15 ratio. In the phenolate ligands, the proton of the phenolic hy­droxy group transfers to the imine N atom. This proton is also involved in an intra­molecular N—H⋯O hydrogen bond that imposes a nearly planar conformation on each ligand, with dihedral angles of 1.75 (4), 3.68 (5) and 3.86 (4)° between the aromatic rings of each ligand.

## Related literature

For related La(III) and Tb(III) complexes, see: Liu *et al.* (2009 ▶); Zhao *et al.* (2007 ▶). For a coordination polymer derived from the same ligand, see: Li *et al.* (2008 ▶). For other complexes of *N*-salicyl­idene­amino acids, see: Burrows & Bailar (1966 ▶). For the synthesis of rare earth complexes with Schiff bases derived from *o*-vanillin and adamantane­amine, see: Zhao *et al.* (2005 ▶) and for chiral lanthanide La(III), Ce(III), Eu(III) complexes with macrocyclic Schiff bases, see: Mazurek & Lisowski (2003 ▶).
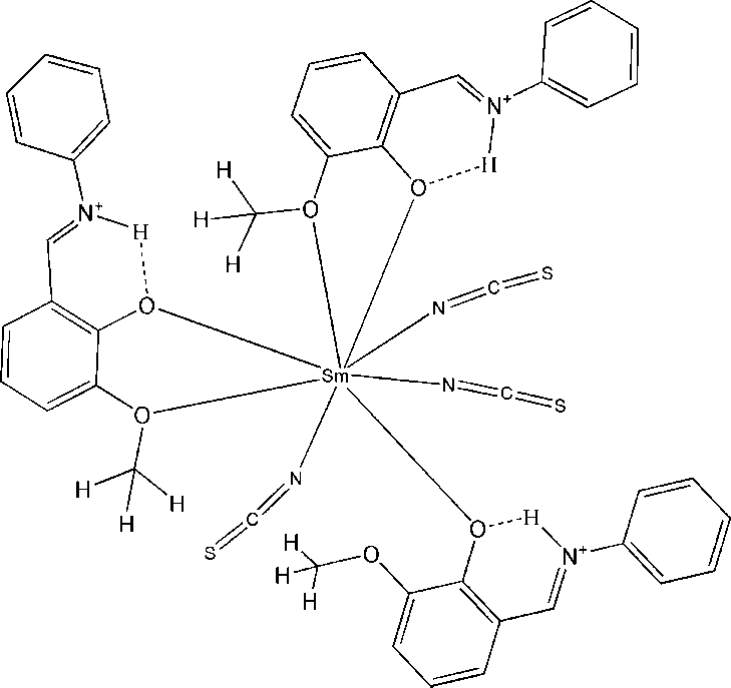


## Experimental

### 

#### Crystal data

[Sm(NCS)_3_(C_14_H_13_NO_2_)_3_]
*M* *_r_* = 1006.35
Orthorhombic, 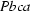

*a* = 19.5821 (13) Å
*b* = 20.3531 (14) Å
*c* = 22.4764 (16) Å
*V* = 8958.1 (11) Å^3^
*Z* = 8
Mo *K*α radiation
μ = 1.51 mm^−1^
*T* = 296 K
0.19 × 0.11 × 0.09 mm

#### Data collection

Bruker APEXII CCD diffractometer
Absorption correction: multi-scan (*SADABS*; Sheldrick, 1996 ▶) *T*_min_ = 0.820, *T*_max_ = 0.879
44977 measured reflections
10202 independent reflections
5925 reflections with *I* > 2σ(*I*)
*R*_int_ = 0.046

#### Refinement

*R*[*F* ^2^ > 2σ(*F* ^2^)] = 0.040
*wR*(*F* ^2^) = 0.077
*S* = 0.99
10202 reflections
562 parameters
H-atom parameters constrained
Δρ_max_ = 0.60 e Å^−3^
Δρ_min_ = −0.48 e Å^−3^

### 

Data collection: *APEX2* (Bruker, 2006 ▶); cell refinement: *SAINT* (Bruker, 2006 ▶); data reduction: *SAINT*; program(s) used to solve structure: *SHELXS97* (Sheldrick, 2008 ▶); program(s) used to refine structure: *SHELXL97* (Sheldrick, 2008 ▶); molecular graphics: *SHELXTL* (Sheldrick, 2008 ▶); software used to prepare material for publication: *SHELXL97*.

## Supplementary Material

Crystal structure: contains datablocks I, global. DOI: 10.1107/S1600536811016205/sj5128sup1.cif

Structure factors: contains datablocks I. DOI: 10.1107/S1600536811016205/sj5128Isup2.hkl

Additional supplementary materials:  crystallographic information; 3D view; checkCIF report

## Figures and Tables

**Table 1:** Selected bond lengths (Å)

Sm1—O4	2.305 (2)
Sm1—O6	2.358 (2)
Sm1—O1	2.391 (2)
Sm1—N3	2.447 (3)
Sm1—N2	2.487 (3)
Sm1—N1	2.499 (3)
Sm1—O5	2.653 (2)
Sm1—O2	2.745 (2)

**Table 2:** Hydrogen-bond geometry (Å, °)

*D*—H⋯*A*	*D*—H	H⋯*A*	*D*⋯*A*	*D*—H⋯*A*
N4—H4*N*⋯O1	0.86	1.83	2.543 (4)	139
N5—H5*N*⋯O6	0.86	1.87	2.573 (3)	131
N6—H6*N*⋯O4	0.86	1.92	2.610 (3)	137

**Fig. 1.**
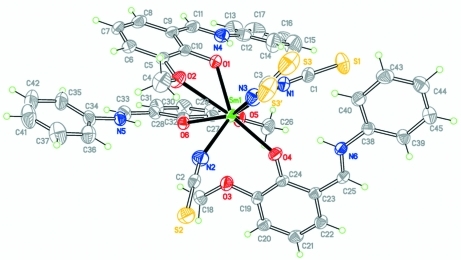
The molecular structure of the title complex, showing the atom-labelling scheme. Displacement ellipsoids are drawn at the 30% probability level.

**Fig. 2.**
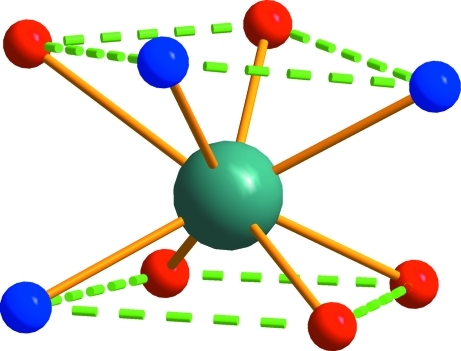
The coordination environment of the samarium(III) atom, showing the square antiprism.

**Fig. 3.**
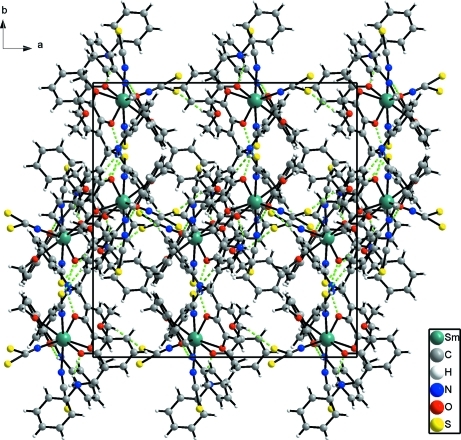
The packing plot of the title compound, showing H-bond interactions (dashed lines).

## supplementary crystallographic information

### Comment

Rare earth complexes with Schiff bases are important in a number of fields such as chemistry and biochemistry owing to their varied characteristics (Zhao *et al.*, 2005; Mazurek & Lisowski, 2003). The complexes prepared by ligands derived from *o*-vanillin have attracted considerable attention for a number of years due to the intriguing biological activities of *o*- vanillin and the convenience of the synthesis of related Schiff bases (Burrows & Bailar, 1966). For these reasons, we have been engaged in the syntheses of new analogous Schiff bases derived from *o*-vanillin and their rare metal complexes (Liu *et al.* 2009; Zhao *et al.*, 2007; Li *et al.*., 2008). Herein, we describe a new Sm^III^ Schiff base complex.

The structure of the title complex is shown in Fig.1, and the coordination environment of Sm^III^ is shown in Fig. 2. The structure of [Sm(NCS)_3_. (C_14_H_13_O_2_N)_3_] (1) contains three (H*L*) ligands and three independent thiocyanate ions. In this complex, the S3 atom of one thiocyanate anion is disordered over two sites (fixed in a 0.85: 0.15 ratio). The Sm^III^ is eight-coordinated by three terminal *N* atoms from three thiocyanate ions and five O atoms from the H*L* ligands in a distorted square antiprismatic geometry. One of the H*L* ligands coordinates in a monodentate fashion to the Sm^III^ ion using oxygen atoms from a deprotonated phenolic hydroxyl group. The other H*L* ligands chelate the Sm^III^ ion through the methoxy O atoms and the deprotonated phenolic hydroxyl O atom. The Sm—O and Sm–N bond distances are listed in Table 1. The Sm—O (phenolic) bond lengths are in the range 2.305 (2) Å - 2.391 (2) Å, and are shorter than those between Sm^III^ and the methoxy O atoms (2.653 (2) Å-2.745 (2) Å), while the Sm—N bonds are 2.447 (3) Å-2.499 (3) Å. The Sm—O (phenolic) and Sm—N bonds are shorter than in the related La(III) complex (Liu *et al.* 2009), which can be attribute to the ionic radii decrease from La(III) to Sm^III^ due to the lanthanide contraction. The H*L* ligands are zwitterionic, with the proton of the phenolic hydroxyl group transferred to the imine *N* atom. This forms an intramolecular N—H···O hydrogen bond and causes the ligands to assume nearly planar conformations. In the crystal, In the crystal structure, molecules are linked by intermolecular C—H···O and C—H···S hydrogen bonds,  Fig 3.

### Experimental

Reagents and solvents used were of commercially available quality and were used without further purification. The Schiff base ligand 2-(phenyliminomethyl)-6- methoxyphenol was synthesized by condensation of *o*-vanillin and aniline. The title compound was synthesized by a traditional method. First, 0.5 mmol Sm(NO_3_)_3_.6H_2_O (dissolved in ethanol) was added dropwise into an ethanol solution with 1.5 mmol H*L*ligand with stirring at room temperature for 2 h to obtain a red solution. 1.5 mmol KNCS (dissolved in ethanol) was then added. The mixture was stirred again for 8 h at room temperature. The resulting solid was filtered out and the solution evaporated yielding red crystals of compound (1) after several days.

### Refinement

The H atoms bonded to C and N atoms were positioned geometrically and refined using a riding model [aliphatic C—H =0.96 Å (*U*_iso_(H) = 1.2*U*_eq_(C)), aromatic C—H = 0.93 Å (*U*_iso_(H) = 1.2*U*_eq_(C)) and N—H = 0.86 Å with *U*_iso_(H) = 1.2*U*_eq_(N)].  The S3 atom of one thiocyanate anion is disordered over two sites and, in the final refinement cycle the occupancies were fixed at 0.85:0.15.

### Crystal data

**Table:**

[Sm(NCS)_3_(C_14_H_13_NO_2_)_3_]	*F*(000) = 4072
*M**_r_* = 1006.35	*D*_x_ = 1.492 Mg m^−^^3^
Orthorhombic, *P**b**c**a*	Mo *K*α radiation, λ = 0.71073 Å
Hall symbol: -P 2ac 2ab	Cell parameters from 8408 reflections
*a* = 19.5821 (13) Å	θ = 1.7–27.4°
*b* = 20.3531 (14) Å	µ = 1.51 mm^−^^1^
*c* = 22.4764 (16) Å	*T* = 296 K
*V* = 8958.1 (11) Å^3^	Block, red
*Z* = 8	0.19 × 0.11 × 0.09 mm

### Data collection

**Table:**

Bruker APEXII CCD diffractometer	10202 independent reflections
Radiation source: fine-focus sealed tube	5925 reflections with *I* > 2σ(*I*)
graphite	*R*_int_ = 0.046
φ and ω scans	θ_max_ = 27.4°, θ_min_ = 1.7°
Absorption correction: multi-scan (*SADABS*; Sheldrick, 1996)	*h* = −25→17
*T*_min_ = 0.820, *T*_max_ = 0.879	*k* = −17→26
44977 measured reflections	*l* = −29→19

### Refinement

**Table:**

Refinement on *F*^2^	Primary atom site location: structure-invariant direct methods
Least-squares matrix: full	Secondary atom site location: difference Fourier map
*R*[*F*^2^ > 2σ(*F*^2^)] = 0.040	Hydrogen site location: inferred from neighbouring sites
*wR*(*F*^2^) = 0.077	H-atom parameters constrained
*S* = 0.99	*w* = 1/[σ^2^(*F*_o_^2^) + (0.0276*P*)^2^*P*] where *P* = (*F*_o_^2^ + 2*F*_c_^2^)/3
10202 reflections	(Δ/σ)_max_ = 0.002
562 parameters	Δρ_max_ = 0.60 e Å^−^^3^
0 restraints	Δρ_min_ = −0.48 e Å^−^^3^

### Special details

**Table:**

Geometry. All e.s.d.'s (except the e.s.d. in the dihedral angle between two l.s. planes) are estimated using the full covariance matrix. The cell e.s.d.'s are taken into account individually in the estimation of e.s.d.'s in distances, angles and torsion angles; correlations between e.s.d.'s in cell parameters are only used when they are defined by crystal symmetry. An approximate (isotropic) treatment of cell e.s.d.'s is used for estimating e.s.d.'s involving l.s. planes.
Refinement. Refinement of *F*^2^ against ALL reflections. The weighted *R*-factor *wR* and goodness of fit *S* are based on *F*^2^, conventional *R*-factors *R* are based on *F*, with *F* set to zero for negative *F*^2^. The threshold expression of *F*^2^ > σ(*F*^2^) is used only for calculating *R*-factors(gt) *etc*. and is not relevant to the choice of reflections for refinement. *R*-factors based on *F*^2^ are statistically about twice as large as those based on *F*, and *R*- factors based on ALL data will be even larger.

### Fractional atomic coordinates and isotropic or equivalent isotropic displacement parameters (Å^2^)

**Table:**

	*x*	*y*	*z*	*U*_iso_*/*U*_eq_	Occ. (<1)
Sm1	0.386756 (8)	0.065497 (8)	0.674038 (7)	0.04009 (7)	
C1	0.38840 (17)	−0.1108 (2)	0.69993 (17)	0.0559 (10)	
C2	0.38077 (16)	0.18885 (17)	0.55898 (17)	0.0472 (9)	
C3	0.2424 (2)	0.01245 (19)	0.58901 (18)	0.0649 (12)	
C4	0.23106 (19)	0.17975 (19)	0.67437 (17)	0.0808 (13)	
H4A	0.1855	0.1693	0.6869	0.121*	
H4B	0.2365	0.1684	0.6332	0.121*	
H4C	0.2391	0.2259	0.6795	0.121*	
C5	0.27647 (16)	0.14943 (18)	0.77058 (17)	0.0470 (9)	
C6	0.24777 (16)	0.20102 (18)	0.8016 (2)	0.0601 (11)	
H6	0.2249	0.2343	0.7815	0.072*	
C7	0.25349 (19)	0.2027 (2)	0.8639 (2)	0.0636 (12)	
H7	0.2345	0.2376	0.8849	0.076*	
C8	0.28607 (19)	0.1547 (2)	0.89360 (19)	0.0645 (11)	
H8	0.2898	0.1571	0.9348	0.077*	
C9	0.31460 (17)	0.10066 (19)	0.86309 (17)	0.0494 (9)	
C10	0.30971 (15)	0.09743 (18)	0.80081 (17)	0.0435 (9)	
C11	0.35044 (19)	0.0510 (2)	0.89350 (18)	0.0596 (11)	
H11	0.3550	0.0546	0.9346	0.072*	
C12	0.4210 (2)	−0.0509 (2)	0.8890 (2)	0.0647 (11)	
C13	0.4511 (2)	−0.0488 (2)	0.9441 (2)	0.0906 (14)	
H13	0.4418	−0.0149	0.9706	0.109*	
C14	0.4343 (3)	−0.1015 (3)	0.8512 (2)	0.0960 (15)	
H14	0.4125	−0.1034	0.8144	0.115*	
C18	0.5674 (2)	0.16708 (17)	0.65924 (17)	0.0792 (13)	
H18A	0.5378	0.1974	0.6794	0.119*	
H18B	0.5886	0.1887	0.6261	0.119*	
H18C	0.6019	0.1518	0.6863	0.119*	
C19	0.56142 (17)	0.06593 (17)	0.60434 (15)	0.0441 (8)	
C20	0.62817 (16)	0.06757 (17)	0.58762 (16)	0.0523 (10)	
H20	0.6559	0.1021	0.5998	0.063*	
C21	0.65513 (17)	0.01726 (19)	0.55211 (16)	0.0569 (10)	
H21	0.7006	0.0190	0.5402	0.068*	
C22	0.61540 (16)	−0.03382 (19)	0.53513 (16)	0.0523 (9)	
H22	0.6336	−0.0670	0.5115	0.063*	
C23	0.54599 (16)	−0.03695 (17)	0.55328 (14)	0.0414 (8)	
C24	0.51804 (16)	0.01387 (17)	0.58791 (15)	0.0428 (9)	
C25	0.50616 (16)	−0.09166 (17)	0.53564 (14)	0.0452 (9)	
H25	0.5263	−0.1231	0.5113	0.054*	
C26	0.53626 (17)	−0.02210 (17)	0.73674 (17)	0.0710 (12)	
H26A	0.5346	−0.0500	0.7712	0.106*	
H26B	0.5818	−0.0055	0.7318	0.106*	
H26C	0.5234	−0.0469	0.7021	0.106*	
C27	0.50429 (16)	0.07603 (17)	0.78924 (16)	0.0442 (9)	
C28	0.47831 (17)	0.18348 (17)	0.82938 (16)	0.0473 (9)	
C29	0.54684 (18)	0.06406 (19)	0.83630 (17)	0.0602 (11)	
H29	0.5698	0.0242	0.8388	0.072*	
C30	0.5558 (2)	0.1110 (2)	0.88003 (18)	0.0734 (12)	
H30	0.5852	0.1025	0.9116	0.088*	
C31	0.5223 (2)	0.1691 (2)	0.87766 (17)	0.0712 (12)	
H31	0.5282	0.1998	0.9079	0.085*	
C32	0.46979 (15)	0.13687 (17)	0.78335 (15)	0.0408 (8)	
C33	0.44407 (19)	0.24441 (18)	0.82642 (17)	0.0592 (10)	
H33	0.4499	0.2740	0.8575	0.071*	
C34	0.36501 (17)	0.31872 (19)	0.7765 (2)	0.0561 (10)	
C35	0.3451 (2)	0.3563 (2)	0.8245 (2)	0.0721 (12)	
H35	0.3588	0.3453	0.8628	0.086*	
C36	0.34359 (19)	0.33435 (19)	0.7203 (2)	0.0698 (12)	
H36	0.3575	0.3089	0.6881	0.084*	
C37	0.3013 (2)	0.3878 (2)	0.7112 (2)	0.0921 (15)	
H37	0.2855	0.3977	0.6732	0.111*	
C38	0.39794 (16)	−0.15331 (17)	0.53783 (14)	0.0436 (9)	
C39	0.41983 (17)	−0.20829 (17)	0.50737 (15)	0.0523 (10)	
H39	0.4647	−0.2117	0.4942	0.063*	
C40	0.33179 (17)	−0.14836 (18)	0.55728 (15)	0.0553 (10)	
H40	0.3175	−0.1110	0.5777	0.066*	
C41	0.2829 (2)	0.4263 (2)	0.7592 (3)	0.0990 (19)	
H41	0.2557	0.4632	0.7535	0.119*	
C42	0.3046 (2)	0.4104 (2)	0.8146 (3)	0.0956 (17)	
H42	0.2919	0.4366	0.8466	0.115*	
C43	0.28665 (18)	−0.1986 (2)	0.54662 (17)	0.0650 (11)	
H43	0.2418	−0.1955	0.5599	0.078*	
C44	0.3076 (2)	−0.2534 (2)	0.51645 (17)	0.0627 (11)	
H44	0.2771	−0.2875	0.5093	0.075*	
C45	0.37362 (19)	−0.25807 (18)	0.49683 (17)	0.0619 (11)	
H45	0.3875	−0.2953	0.4761	0.074*	
N1	0.37935 (14)	−0.05526 (14)	0.69309 (14)	0.0592 (9)	
N2	0.38159 (15)	0.15884 (15)	0.60278 (15)	0.0626 (9)	
N3	0.28872 (16)	0.03283 (15)	0.61340 (14)	0.0638 (9)	
N4	0.37735 (15)	0.00056 (16)	0.86737 (14)	0.0589 (9)	
H4N	0.3672	−0.0028	0.8303	0.071*	
N5	0.40494 (13)	0.26107 (13)	0.78251 (13)	0.0496 (8)	
H5N	0.4029	0.2337	0.7534	0.059*	
N6	0.44248 (13)	−0.10033 (13)	0.55171 (11)	0.0451 (7)	
H6N	0.4253	−0.0699	0.5736	0.054*	
O1	0.33589 (11)	0.04926 (10)	0.76964 (9)	0.0477 (6)	
O2	0.27958 (11)	0.14291 (12)	0.70981 (11)	0.0606 (7)	
O3	0.52826 (11)	0.11233 (12)	0.63829 (11)	0.0593 (7)	
O4	0.45412 (10)	0.01339 (10)	0.60439 (10)	0.0506 (6)	
O5	0.48975 (11)	0.03197 (11)	0.74444 (11)	0.0536 (6)	
O6	0.43125 (10)	0.14810 (9)	0.73654 (9)	0.0413 (5)	
S1	0.40327 (6)	−0.18882 (5)	0.70832 (6)	0.0935 (4)	
S2	0.37988 (5)	0.23007 (5)	0.49660 (5)	0.0640 (3)	
S3	0.17603 (7)	−0.02499 (10)	0.55935 (7)	0.0962 (5)	0.85
S3'	0.1863 (4)	0.0303 (5)	0.5308 (4)	0.081 (3)	0.15
C17	0.4967 (3)	−0.0997 (3)	0.9590 (3)	0.1057 (18)	
H17	0.5171	−0.0999	0.9963	0.127*	
C16	0.5111 (3)	−0.1476 (3)	0.9205 (3)	0.112 (2)	
H16	0.5428	−0.1797	0.9305	0.134*	
C15	0.4796 (3)	−0.1498 (3)	0.8670 (3)	0.118 (2)	
H15	0.4888	−0.1841	0.8409	0.142*	

### Atomic displacement parameters (Å^2^)

**Table:**

	*U*^11^	*U*^22^	*U*^33^	*U*^12^	*U*^13^	*U*^23^
Sm1	0.04677 (11)	0.04036 (12)	0.03315 (11)	0.00306 (9)	0.00068 (9)	−0.00664 (9)
C1	0.057 (2)	0.057 (3)	0.054 (3)	0.002 (2)	−0.001 (2)	−0.011 (2)
C2	0.043 (2)	0.048 (2)	0.051 (3)	0.0075 (18)	0.0031 (19)	−0.010 (2)
C3	0.057 (3)	0.077 (3)	0.060 (3)	0.015 (2)	−0.004 (2)	−0.023 (2)
C4	0.072 (3)	0.094 (3)	0.077 (3)	0.024 (3)	−0.020 (2)	0.003 (3)
C5	0.040 (2)	0.053 (2)	0.048 (3)	−0.0033 (19)	0.0100 (18)	−0.006 (2)
C6	0.048 (2)	0.056 (3)	0.076 (3)	0.000 (2)	0.018 (2)	−0.005 (2)
C7	0.060 (3)	0.059 (3)	0.072 (4)	−0.008 (2)	0.030 (2)	−0.026 (3)
C8	0.060 (3)	0.078 (3)	0.055 (3)	−0.016 (2)	0.015 (2)	−0.022 (3)
C9	0.050 (2)	0.058 (3)	0.041 (3)	−0.006 (2)	0.0101 (19)	−0.004 (2)
C10	0.040 (2)	0.047 (2)	0.044 (2)	−0.0097 (18)	0.0076 (17)	−0.009 (2)
C11	0.060 (2)	0.075 (3)	0.043 (3)	−0.012 (2)	0.010 (2)	−0.007 (2)
C12	0.069 (3)	0.078 (3)	0.048 (3)	−0.009 (3)	0.000 (2)	0.021 (3)
C13	0.086 (3)	0.117 (4)	0.069 (4)	−0.003 (3)	−0.008 (3)	0.006 (3)
C14	0.144 (5)	0.076 (4)	0.069 (4)	0.017 (4)	−0.001 (3)	0.001 (3)
C18	0.099 (3)	0.056 (3)	0.083 (3)	−0.028 (2)	0.024 (3)	−0.019 (2)
C19	0.049 (2)	0.045 (2)	0.038 (2)	0.001 (2)	−0.0007 (17)	−0.0071 (19)
C20	0.045 (2)	0.061 (3)	0.051 (3)	−0.0131 (19)	−0.0001 (17)	−0.005 (2)
C21	0.039 (2)	0.071 (3)	0.061 (3)	−0.003 (2)	0.0064 (19)	−0.002 (2)
C22	0.044 (2)	0.057 (2)	0.056 (3)	0.003 (2)	0.0033 (19)	−0.008 (2)
C23	0.0390 (19)	0.049 (2)	0.036 (2)	−0.0021 (18)	0.0006 (16)	−0.0018 (19)
C24	0.042 (2)	0.051 (2)	0.035 (2)	−0.0018 (19)	0.0049 (17)	−0.0018 (19)
C25	0.045 (2)	0.050 (2)	0.040 (2)	0.0088 (19)	0.0064 (17)	−0.0029 (19)
C26	0.072 (3)	0.064 (3)	0.077 (3)	0.028 (2)	0.001 (2)	0.000 (2)
C27	0.0412 (19)	0.050 (2)	0.042 (2)	0.0030 (18)	0.0001 (17)	0.003 (2)
C28	0.055 (2)	0.046 (2)	0.041 (2)	−0.0047 (19)	−0.0063 (19)	0.000 (2)
C29	0.057 (2)	0.064 (3)	0.060 (3)	0.000 (2)	−0.012 (2)	0.009 (2)
C30	0.079 (3)	0.082 (3)	0.059 (3)	0.002 (3)	−0.029 (2)	0.009 (3)
C31	0.093 (3)	0.074 (3)	0.046 (3)	−0.003 (3)	−0.029 (2)	−0.013 (2)
C32	0.0366 (19)	0.046 (2)	0.039 (2)	−0.0080 (17)	0.0003 (17)	0.0024 (19)
C33	0.069 (3)	0.055 (3)	0.053 (3)	−0.008 (2)	−0.008 (2)	−0.008 (2)
C34	0.051 (2)	0.041 (2)	0.076 (3)	−0.0066 (19)	−0.003 (2)	−0.007 (2)
C35	0.072 (3)	0.057 (3)	0.087 (3)	0.007 (2)	−0.001 (2)	−0.024 (3)
C36	0.067 (3)	0.062 (3)	0.080 (4)	0.010 (2)	−0.008 (3)	−0.006 (3)
C37	0.083 (3)	0.079 (4)	0.115 (5)	0.011 (3)	−0.036 (3)	0.000 (3)
C38	0.044 (2)	0.048 (2)	0.038 (2)	−0.0058 (18)	−0.0022 (16)	−0.0033 (18)
C39	0.049 (2)	0.048 (2)	0.060 (3)	−0.0038 (19)	−0.0063 (19)	−0.006 (2)
C40	0.048 (2)	0.063 (3)	0.055 (3)	−0.005 (2)	0.0050 (19)	−0.011 (2)
C41	0.067 (3)	0.061 (3)	0.169 (6)	0.012 (2)	−0.027 (4)	−0.024 (4)
C42	0.073 (3)	0.071 (4)	0.142 (6)	0.006 (3)	−0.005 (3)	−0.044 (4)
C43	0.049 (2)	0.084 (3)	0.062 (3)	−0.014 (2)	0.003 (2)	−0.006 (3)
C44	0.062 (3)	0.067 (3)	0.059 (3)	−0.017 (2)	−0.019 (2)	0.003 (2)
C45	0.067 (3)	0.049 (3)	0.069 (3)	−0.001 (2)	−0.009 (2)	−0.007 (2)
N1	0.069 (2)	0.046 (2)	0.063 (2)	0.0055 (17)	0.0125 (16)	−0.0055 (17)
N2	0.070 (2)	0.066 (2)	0.052 (2)	0.0013 (17)	−0.0009 (18)	0.0064 (19)
N3	0.063 (2)	0.069 (2)	0.059 (2)	−0.0020 (19)	−0.0075 (18)	−0.0119 (19)
N4	0.067 (2)	0.070 (2)	0.040 (2)	−0.0049 (19)	0.0010 (17)	0.0039 (19)
N5	0.0502 (18)	0.0411 (19)	0.057 (2)	−0.0014 (15)	−0.0027 (16)	−0.0063 (16)
N6	0.0447 (16)	0.0482 (18)	0.0425 (19)	−0.0017 (15)	0.0058 (14)	−0.0098 (15)
O1	0.0552 (14)	0.0450 (14)	0.0430 (15)	−0.0002 (12)	0.0069 (12)	−0.0081 (12)
O2	0.0634 (16)	0.0774 (18)	0.0409 (17)	0.0180 (14)	−0.0021 (13)	−0.0037 (14)
O3	0.0580 (15)	0.0552 (16)	0.0645 (18)	−0.0069 (14)	0.0082 (13)	−0.0191 (14)
O4	0.0408 (13)	0.0598 (16)	0.0513 (16)	−0.0045 (12)	0.0109 (11)	−0.0150 (13)
O5	0.0544 (14)	0.0504 (15)	0.0559 (17)	0.0158 (13)	−0.0083 (13)	−0.0085 (14)
O6	0.0449 (13)	0.0418 (13)	0.0371 (14)	0.0018 (11)	−0.0036 (11)	−0.0021 (12)
S1	0.1156 (10)	0.0449 (7)	0.1200 (11)	0.0103 (6)	−0.0151 (8)	−0.0014 (7)
S2	0.0645 (6)	0.0674 (7)	0.0601 (7)	0.0146 (5)	0.0041 (5)	0.0142 (6)
S3	0.0590 (9)	0.1447 (15)	0.0849 (13)	0.0049 (11)	−0.0177 (8)	−0.0495 (12)
S3'	0.057 (5)	0.120 (7)	0.067 (6)	0.022 (5)	−0.031 (4)	−0.042 (6)
C17	0.088 (4)	0.134 (5)	0.095 (5)	0.009 (4)	−0.019 (3)	0.049 (4)
C16	0.113 (4)	0.103 (5)	0.120 (6)	0.024 (4)	0.029 (4)	0.049 (4)
C15	0.163 (6)	0.088 (4)	0.104 (5)	0.037 (4)	0.006 (4)	0.025 (4)

### Geometric parameters (Å, °)

**Table:**

Sm1—O4	2.305 (2)	C23—C25	1.416 (4)
Sm1—O6	2.358 (2)	C24—O4	1.306 (3)
Sm1—O1	2.391 (2)	C25—N6	1.310 (3)
Sm1—N3	2.447 (3)	C25—H25	0.9300
Sm1—N2	2.487 (3)	C26—O5	1.439 (3)
Sm1—N1	2.499 (3)	C26—H26A	0.9600
Sm1—O5	2.653 (2)	C26—H26B	0.9600
Sm1—O2	2.745 (2)	C26—H26C	0.9600
C1—N1	1.155 (4)	C27—C29	1.368 (4)
C1—S1	1.625 (4)	C27—O5	1.378 (4)
C2—N2	1.159 (4)	C27—C32	1.417 (4)
C2—S2	1.634 (4)	C28—C33	1.411 (4)
C3—N3	1.139 (4)	C28—C32	1.414 (4)
C3—S3	1.647 (4)	C28—C31	1.415 (4)
C3—S3'	1.746 (8)	C29—C30	1.381 (5)
C4—O2	1.449 (4)	C29—H29	0.9300
C4—H4A	0.9600	C30—C31	1.355 (5)
C4—H4B	0.9600	C30—H30	0.9300
C4—H4C	0.9600	C31—H31	0.9300
C5—O2	1.374 (4)	C32—O6	1.315 (3)
C5—C6	1.380 (4)	C33—N5	1.295 (4)
C5—C10	1.416 (4)	C33—H33	0.9300
C6—C7	1.405 (5)	C34—C36	1.367 (5)
C6—H6	0.9300	C34—C35	1.379 (5)
C7—C8	1.345 (5)	C34—N5	1.416 (4)
C7—H7	0.9300	C35—C42	1.374 (5)
C8—C9	1.411 (5)	C35—H35	0.9300
C8—H8	0.9300	C36—C37	1.382 (5)
C9—C10	1.405 (4)	C36—H36	0.9300
C9—C11	1.407 (5)	C37—C41	1.382 (6)
C10—O1	1.310 (4)	C37—H37	0.9300
C11—N4	1.295 (4)	C38—C40	1.371 (4)
C11—H11	0.9300	C38—C39	1.380 (4)
C12—C14	1.361 (5)	C38—N6	1.422 (4)
C12—C13	1.372 (5)	C39—C45	1.379 (4)
C12—N4	1.436 (5)	C39—H39	0.9300
C13—C17	1.408 (6)	C40—C43	1.373 (4)
C13—H13	0.9300	C40—H40	0.9300
C14—C15	1.371 (6)	C41—C42	1.355 (6)
C14—H14	0.9300	C41—H41	0.9300
C18—O3	1.432 (4)	C42—H42	0.9300
C18—H18A	0.9600	C43—C44	1.369 (5)
C18—H18B	0.9600	C43—H43	0.9300
C18—H18C	0.9600	C44—C45	1.369 (4)
C19—C20	1.360 (4)	C44—H44	0.9300
C19—O3	1.377 (3)	C45—H45	0.9300
C19—C24	1.407 (4)	N4—H4N	0.8600
C20—C21	1.402 (4)	N5—H5N	0.8600
C20—H20	0.9300	N6—H6N	0.8600
C21—C22	1.354 (4)	C17—C16	1.333 (6)
C21—H21	0.9300	C17—H17	0.9300
C22—C23	1.421 (4)	C16—C15	1.351 (7)
C22—H22	0.9300	C16—H16	0.9300
C23—C24	1.405 (4)	C15—H15	0.9300
			
O4—Sm1—O6	121.41 (7)	C23—C25—H25	118.2
O4—Sm1—O1	141.71 (7)	O5—C26—H26A	109.5
O6—Sm1—O1	73.57 (7)	O5—C26—H26B	109.5
O4—Sm1—N3	86.89 (9)	H26A—C26—H26B	109.5
O6—Sm1—N3	144.66 (9)	O5—C26—H26C	109.5
O1—Sm1—N3	97.83 (9)	H26A—C26—H26C	109.5
O4—Sm1—N2	86.41 (9)	H26B—C26—H26C	109.5
O6—Sm1—N2	81.60 (9)	C29—C27—O5	125.1 (3)
O1—Sm1—N2	131.86 (9)	C29—C27—C32	121.2 (4)
N3—Sm1—N2	79.45 (10)	O5—C27—C32	113.7 (3)
O4—Sm1—N1	72.37 (9)	C33—C28—C32	119.9 (3)
O6—Sm1—N1	128.36 (9)	C33—C28—C31	120.4 (4)
O1—Sm1—N1	71.69 (8)	C32—C28—C31	119.6 (3)
N3—Sm1—N1	77.45 (10)	C27—C29—C30	120.3 (4)
N2—Sm1—N1	149.24 (10)	C27—C29—H29	119.9
O4—Sm1—O5	81.47 (7)	C30—C29—H29	119.9
O6—Sm1—O5	63.07 (7)	C31—C30—C29	121.0 (4)
O1—Sm1—O5	75.23 (7)	C31—C30—H30	119.5
N3—Sm1—O5	149.21 (9)	C29—C30—H30	119.5
N2—Sm1—O5	127.70 (8)	C30—C31—C28	120.3 (4)
N1—Sm1—O5	71.88 (9)	C30—C31—H31	119.8
O4—Sm1—O2	154.22 (8)	C28—C31—H31	119.8
O6—Sm1—O2	72.46 (7)	O6—C32—C28	122.5 (3)
O1—Sm1—O2	59.84 (7)	O6—C32—C27	120.0 (3)
N3—Sm1—O2	73.69 (9)	C28—C32—C27	117.5 (3)
N2—Sm1—O2	73.68 (9)	N5—C33—C28	123.2 (3)
N1—Sm1—O2	118.03 (8)	N5—C33—H33	118.4
O5—Sm1—O2	123.66 (7)	C28—C33—H33	118.4
N1—C1—S1	178.2 (4)	C36—C34—C35	120.4 (4)
N2—C2—S2	179.1 (3)	C36—C34—N5	116.8 (4)
N3—C3—S3	172.8 (4)	C35—C34—N5	122.7 (4)
N3—C3—S3'	141.8 (5)	C42—C35—C34	118.8 (4)
S3—C3—S3'	45.4 (3)	C42—C35—H35	120.6
O2—C4—H4A	109.5	C34—C35—H35	120.6
O2—C4—H4B	109.5	C34—C36—C37	120.3 (4)
H4A—C4—H4B	109.5	C34—C36—H36	119.9
O2—C4—H4C	109.5	C37—C36—H36	119.9
H4A—C4—H4C	109.5	C41—C37—C36	119.1 (5)
H4B—C4—H4C	109.5	C41—C37—H37	120.4
O2—C5—C6	126.4 (4)	C36—C37—H37	120.4
O2—C5—C10	112.6 (3)	C40—C38—C39	120.7 (3)
C6—C5—C10	120.9 (4)	C40—C38—N6	117.0 (3)
C5—C6—C7	119.3 (4)	C39—C38—N6	122.2 (3)
C5—C6—H6	120.3	C45—C39—C38	118.5 (3)
C7—C6—H6	120.3	C45—C39—H39	120.7
C8—C7—C6	121.0 (4)	C38—C39—H39	120.7
C8—C7—H7	119.5	C38—C40—C43	119.9 (3)
C6—C7—H7	119.5	C38—C40—H40	120.1
C7—C8—C9	120.9 (4)	C43—C40—H40	120.1
C7—C8—H8	119.6	C42—C41—C37	120.0 (5)
C9—C8—H8	119.6	C42—C41—H41	120.0
C10—C9—C11	119.0 (4)	C37—C41—H41	120.0
C10—C9—C8	119.6 (4)	C41—C42—C35	121.4 (5)
C11—C9—C8	121.4 (4)	C41—C42—H42	119.3
O1—C10—C9	122.8 (4)	C35—C42—H42	119.3
O1—C10—C5	118.9 (3)	C44—C43—C40	120.0 (4)
C9—C10—C5	118.3 (4)	C44—C43—H43	120.0
N4—C11—C9	123.5 (4)	C40—C43—H43	120.0
N4—C11—H11	118.2	C43—C44—C45	120.0 (4)
C9—C11—H11	118.2	C43—C44—H44	120.0
C14—C12—C13	120.3 (5)	C45—C44—H44	120.0
C14—C12—N4	117.0 (4)	C44—C45—C39	120.9 (4)
C13—C12—N4	122.6 (5)	C44—C45—H45	119.5
C12—C13—C17	117.6 (5)	C39—C45—H45	119.5
C12—C13—H13	121.2	C1—N1—Sm1	167.7 (3)
C17—C13—H13	121.2	C2—N2—Sm1	161.9 (3)
C12—C14—C15	120.3 (5)	C3—N3—Sm1	173.1 (3)
C12—C14—H14	119.9	C11—N4—C12	131.8 (4)
C15—C14—H14	119.9	C11—N4—H4N	114.1
O3—C18—H18A	109.5	C12—N4—H4N	114.1
O3—C18—H18B	109.5	C33—N5—C34	128.1 (3)
H18A—C18—H18B	109.5	C33—N5—H5N	116.0
O3—C18—H18C	109.5	C34—N5—H5N	116.0
H18A—C18—H18C	109.5	C25—N6—C38	128.7 (3)
H18B—C18—H18C	109.5	C25—N6—H6N	115.6
C20—C19—O3	126.1 (3)	C38—N6—H6N	115.6
C20—C19—C24	121.7 (3)	C10—O1—Sm1	122.7 (2)
O3—C19—C24	112.2 (3)	C5—O2—C4	117.9 (3)
C19—C20—C21	120.1 (3)	C5—O2—Sm1	112.4 (2)
C19—C20—H20	120.0	C4—O2—Sm1	129.6 (2)
C21—C20—H20	120.0	C19—O3—C18	117.6 (3)
C22—C21—C20	120.4 (3)	C24—O4—Sm1	137.6 (2)
C22—C21—H21	119.8	C27—O5—C26	117.1 (3)
C20—C21—H21	119.8	C27—O5—Sm1	115.19 (18)
C21—C22—C23	120.2 (3)	C26—O5—Sm1	127.3 (2)
C21—C22—H22	119.9	C32—O6—Sm1	124.4 (2)
C23—C22—H22	119.9	C16—C17—C13	121.2 (6)
C24—C23—C25	121.3 (3)	C16—C17—H17	119.4
C24—C23—C22	119.9 (3)	C13—C17—H17	119.4
C25—C23—C22	118.8 (3)	C17—C16—C15	120.3 (6)
O4—C24—C23	121.7 (3)	C17—C16—H16	119.9
O4—C24—C19	120.7 (3)	C15—C16—H16	119.9
C23—C24—C19	117.7 (3)	C16—C15—C14	120.2 (6)
N6—C25—C23	123.6 (3)	C16—C15—H15	119.9
N6—C25—H25	118.2	C14—C15—H15	119.9
			
O2—C5—C6—C7	−175.1 (3)	O5—Sm1—N2—C2	106.9 (9)
C10—C5—C6—C7	1.9 (5)	O2—Sm1—N2—C2	−132.8 (9)
C5—C6—C7—C8	−0.4 (5)	C9—C11—N4—C12	172.7 (3)
C6—C7—C8—C9	−0.9 (5)	C14—C12—N4—C11	172.7 (4)
C7—C8—C9—C10	0.8 (5)	C13—C12—N4—C11	−10.2 (6)
C7—C8—C9—C11	178.2 (3)	C28—C33—N5—C34	175.8 (3)
C11—C9—C10—O1	1.5 (5)	C36—C34—N5—C33	161.9 (4)
C8—C9—C10—O1	179.0 (3)	C35—C34—N5—C33	−21.3 (5)
C11—C9—C10—C5	−176.9 (3)	C23—C25—N6—C38	−178.8 (3)
C8—C9—C10—C5	0.6 (5)	C40—C38—N6—C25	−175.9 (3)
O2—C5—C10—O1	−3.0 (4)	C39—C38—N6—C25	5.5 (5)
C6—C5—C10—O1	179.6 (3)	C9—C10—O1—Sm1	−143.4 (2)
O2—C5—C10—C9	175.4 (3)	C5—C10—O1—Sm1	35.0 (4)
C6—C5—C10—C9	−1.9 (5)	O4—Sm1—O1—C10	166.8 (2)
C10—C9—C11—N4	−2.7 (5)	O6—Sm1—O1—C10	46.5 (2)
C8—C9—C11—N4	179.9 (3)	N3—Sm1—O1—C10	−98.3 (2)
C14—C12—C13—C17	1.3 (6)	N2—Sm1—O1—C10	−15.5 (3)
N4—C12—C13—C17	−175.7 (4)	N1—Sm1—O1—C10	−172.3 (2)
C13—C12—C14—C15	−2.1 (7)	O5—Sm1—O1—C10	112.3 (2)
N4—C12—C14—C15	175.0 (4)	O2—Sm1—O1—C10	−32.4 (2)
O3—C19—C20—C21	−179.1 (3)	C6—C5—O2—C4	−21.7 (5)
C24—C19—C20—C21	1.3 (5)	C10—C5—O2—C4	161.1 (3)
C19—C20—C21—C22	−1.2 (5)	C6—C5—O2—Sm1	154.3 (3)
C20—C21—C22—C23	−0.1 (5)	C10—C5—O2—Sm1	−22.9 (3)
C21—C22—C23—C24	1.4 (5)	O4—Sm1—O2—C5	179.93 (19)
C21—C22—C23—C25	−178.9 (3)	O6—Sm1—O2—C5	−53.0 (2)
C25—C23—C24—O4	−1.2 (5)	O1—Sm1—O2—C5	27.8 (2)
C22—C23—C24—O4	178.6 (3)	N3—Sm1—O2—C5	137.3 (2)
C25—C23—C24—C19	179.0 (3)	N2—Sm1—O2—C5	−139.2 (2)
C22—C23—C24—C19	−1.3 (5)	N1—Sm1—O2—C5	71.7 (2)
C20—C19—C24—O4	−179.9 (3)	O5—Sm1—O2—C5	−14.4 (2)
O3—C19—C24—O4	0.4 (4)	O4—Sm1—O2—C4	−4.7 (4)
C20—C19—C24—C23	0.0 (5)	O6—Sm1—O2—C4	122.3 (3)
O3—C19—C24—C23	−179.7 (3)	O1—Sm1—O2—C4	−156.8 (3)
C24—C23—C25—N6	−2.7 (5)	N3—Sm1—O2—C4	−47.3 (3)
C22—C23—C25—N6	177.6 (3)	N2—Sm1—O2—C4	36.2 (3)
O5—C27—C29—C30	−177.5 (3)	N1—Sm1—O2—C4	−113.0 (3)
C32—C27—C29—C30	1.5 (5)	O5—Sm1—O2—C4	161.0 (3)
C27—C29—C30—C31	0.7 (6)	C20—C19—O3—C18	−1.3 (5)
C29—C30—C31—C28	−1.3 (6)	C24—C19—O3—C18	178.4 (3)
C33—C28—C31—C30	−179.1 (4)	C23—C24—O4—Sm1	163.2 (2)
C32—C28—C31—C30	−0.4 (5)	C19—C24—O4—Sm1	−16.9 (5)
C33—C28—C32—O6	0.8 (5)	O6—Sm1—O4—C24	3.3 (3)
C31—C28—C32—O6	−178.0 (3)	O1—Sm1—O4—C24	−100.5 (3)
C33—C28—C32—C27	−178.8 (3)	N3—Sm1—O4—C24	160.8 (3)
C31—C28—C32—C27	2.5 (5)	N2—Sm1—O4—C24	81.2 (3)
C29—C27—C32—O6	177.4 (3)	N1—Sm1—O4—C24	−121.4 (3)
O5—C27—C32—O6	−3.5 (4)	O5—Sm1—O4—C24	−47.8 (3)
C29—C27—C32—C28	−3.1 (5)	O2—Sm1—O4—C24	120.2 (3)
O5—C27—C32—C28	176.1 (3)	C29—C27—O5—C26	−18.8 (5)
C32—C28—C33—N5	−1.0 (5)	C32—C27—O5—C26	162.1 (3)
C31—C28—C33—N5	177.8 (3)	C29—C27—O5—Sm1	168.3 (3)
C36—C34—C35—C42	−1.4 (6)	C32—C27—O5—Sm1	−10.7 (3)
N5—C34—C35—C42	−178.0 (3)	O4—Sm1—O5—C27	145.8 (2)
C35—C34—C36—C37	−0.4 (6)	O6—Sm1—O5—C27	13.97 (19)
N5—C34—C36—C37	176.4 (3)	O1—Sm1—O5—C27	−64.8 (2)
C34—C36—C37—C41	2.1 (6)	N3—Sm1—O5—C27	−145.2 (2)
C40—C38—C39—C45	0.1 (5)	N2—Sm1—O5—C27	67.1 (2)
N6—C38—C39—C45	178.6 (3)	N1—Sm1—O5—C27	−140.0 (2)
C39—C38—C40—C43	0.2 (5)	O2—Sm1—O5—C27	−27.9 (2)
N6—C38—C40—C43	−178.4 (3)	O4—Sm1—O5—C26	−26.2 (2)
C36—C37—C41—C42	−1.9 (7)	O6—Sm1—O5—C26	−158.1 (3)
C37—C41—C42—C35	0.1 (8)	O1—Sm1—O5—C26	123.1 (2)
C34—C35—C42—C41	1.5 (7)	N3—Sm1—O5—C26	42.8 (3)
C38—C40—C43—C44	−0.2 (5)	N2—Sm1—O5—C26	−104.9 (3)
C40—C43—C44—C45	−0.1 (6)	N1—Sm1—O5—C26	47.9 (2)
C43—C44—C45—C39	0.5 (6)	O2—Sm1—O5—C26	160.0 (2)
C38—C39—C45—C44	−0.5 (5)	C28—C32—O6—Sm1	−160.2 (2)
O4—Sm1—N1—C1	41.7 (14)	C27—C32—O6—Sm1	19.3 (4)
O6—Sm1—N1—C1	−74.8 (14)	O4—Sm1—O6—C32	−76.9 (2)
O1—Sm1—N1—C1	−124.8 (14)	O1—Sm1—O6—C32	64.2 (2)
N3—Sm1—N1—C1	132.4 (14)	N3—Sm1—O6—C32	144.4 (2)
N2—Sm1—N1—C1	90.2 (14)	N2—Sm1—O6—C32	−157.5 (2)
O5—Sm1—N1—C1	−44.9 (14)	N1—Sm1—O6—C32	14.9 (2)
O2—Sm1—N1—C1	−164.0 (14)	O5—Sm1—O6—C32	−17.2 (2)
O4—Sm1—N2—C2	30.6 (9)	O2—Sm1—O6—C32	127.1 (2)
O6—Sm1—N2—C2	153.1 (9)	C12—C13—C17—C16	1.2 (7)
O1—Sm1—N2—C2	−148.0 (9)	C13—C17—C16—C15	−2.9 (9)
N3—Sm1—N2—C2	−56.9 (9)	C17—C16—C15—C14	2.0 (9)
N1—Sm1—N2—C2	−15.1 (10)	C12—C14—C15—C16	0.5 (8)

### Hydrogen-bond geometry (Å, °)

**Table:**

*D*—H···*A*	*D*—H	H···*A*	*D*···*A*	*D*—H···*A*
N4—H4N···O1	0.86	1.83	2.543 (4)	139
N5—H5N···O6	0.86	1.87	2.573 (3)	131
N6—H6N···O4	0.86	1.92	2.610 (3)	137
